# ‘Windows of opportunity’: exploring the relationship between social media and plastic policies during the COVID-19 Pandemic

**DOI:** 10.1007/s11077-022-09479-x

**Published:** 2022-11-15

**Authors:** Joanna Vince, Estelle Praet, John Schofield, Kathy Townsend

**Affiliations:** 1 School of Social Sciences, University of Tasmania, Launceston, TAS 7250 Australia; 2 Centre for Marine Socio-Ecology, University of Tasmania, Hobart, TAS 7250 Australia; 3 Department of Archaeology, University of York, York, UK; 4 School of Science, Technology, and Engineering, University of the Sunshine Coast, Sippy Downs, QLD Australia

**Keywords:** Crisis, Plastic pollution, COVID-19, Agenda setting, Entrepreneurs, Social media, Media, Archaeology, Ecology

## Abstract

Plastic pollution has reached a crisis point due to ineffective waste management, an over-reliance on single-use plastic items and a lack of suitable plastic alternatives. The COVID-19 Pandemic has seen a dramatic increase in the use of single-use plastics including ‘COVID waste’ in the form of items specifically intended to help stop the spread of disease. Many governments have utilised COVID-19 as a window of opportunity to reverse, postpone or remove plastic policies off agendas ostensibly in order to ‘flatten the curve’ of COVID-19 cases. In this paper, we use novel methods of social media analysis relating to three regions (USA, Mexico and Australia) to suggest that health and hygiene were not the only reasons governments utilised this window of opportunity to change plastic policies. Beyond the influence of social media on the plastics agenda, our results highlight the potential of social media as a tool to analyse public reactions to government decisions that can be influenced by industry pressure and a broader political agenda, while not necessarily following responses to consumer behaviour.

## Introduction

Plastic waste pollution has become a large and complex global governance problem to solve. Often described as a ‘wicked problem’ (Landon-Lane, 2018; Vince & Stoett, 2018) and a ‘creeping crisis’ (Mæland & Staupe‐Delgado, 2020), plastic pollution has far-reaching consequences—it is found in terrestrial and marine environments, from the Swiss Alps (Bergmann et al., 2019) to the deep ocean (Chiba et al., 2018), and in the most remote places in the world such as Henderson Island (Lavers & Bond, 2017). As a material, plastic is culturally embedded in society (da Costa et al., 2020) through its practicality and purposefulness to the extent that it is now considered across disciplines, including archaeology, as a key signature of a Plastic Age (Pétursdóttir, 2017; Schofield et al., 2021; Thompson et al., 2009) or, as an epoch, the Plasticene (Ross, 2018).

The impacts of plastic pollution are diverse and widespread. It has an estimated social and environmental cost of US$3.7 trillion each year (DeWit et al., 2021). It has been linked to climate change with plastic degradation contributing methane and ethylene to the atmosphere (Royer et al., 2018). Despite a better understanding of the environmental and societal problems caused by plastic over the last few decades (e.g. cost in Forrest et al., 2019; link to climate change in Stoett & Vince, 2021; impact on human health in Flaws et al., 2020), there has been more plastic in the environment, not less. This is acknowledged on a global scale with the United Nations (UN) Environment Assembly passing a resolution in March 2022 where members agreed that by 2024 they will have developed an international legally binding agreement to “End Plastic Pollution” (Draft Res of 2 March 2022). Nation states have recognised that the plastic issue is something that needs to be addressed through policy in their jurisdictions. Yet, solutions are slow to be placed onto political agendas.

The COVID-19 Pandemic is hitting the world severely, leading to the death of more than 6.5 million people (at the time of writing) (World Health Organisation, 2022) resulting in an overwhelmed and exhausted global healthcare system. This focussing event has resulted in governments across the world prioritising COVID-19 on their political agendas and implementing health measures needed to ‘flatten the curve’ of the Pandemic. This ‘health and hygiene’ approach has provided some governments the opportunity to remove plastic policies from their agendas with little consultation or limited notification (da Costa et al., 2020; Prata et al., 2020; Silva et al., 2020).

Social media have been utilised by governments, decision makers, policy entrepreneurs, industry and the general public to share information on the Pandemic and the increase in the use of single-use plastics. Social and mainstream media have had an important role in educating the public about COVID-19 and being the linkage between governments and the general public about health and safety, as they would in other crises (Friedman et al., 2019). Social media have also been instrumental in raising awareness of the plastic crisis, focussing attention on the growing amount of ‘COVID waste’, stimulating behavioural change to reduce plastic consumption and providing public pressure to drive the transition from a linear to a circular economy (da Costa et al., 2020).

In this paper we offer reflections about the occurrence of a window of opportunity due to the COVID-19 Pandemic to change plastic policies driven by industry pressure, responses to consumer behaviour and/or political pressure. We begin by examining social media and COVID-19 as a window of opportunity for policy entrepreneurs to engage in policy change. This is followed by an overview of the Pandemic’s influence on the plastic agenda. Utilising a social media analysis approach we then examine three case studies to analyse the key drivers within government decision making. The first case study analyses industry pressure in the USA where pro-plastic entrepreneurs are key actors in plastic policy decision making. The second case study examines the politics behind plastic policies in Mexico. And lastly, the third case study analyses consumer behaviour in Australia and government non-decision making on plastic pollution issues.

### Social media and COVID-19 as a window of opportunity

In times of crisis, focussing events or external unexpected shocks (Birkland, 1998), such as COVID-19, open policy windows to initiate change (Kingdon, 1995; Michaels et al., 2006). Media are particularly quick to respond to focussing events and can contribute to how long an event is considered important and eventually to the size of policy windows. Policy entrepreneurs are often the people to drive policy change, and these include people from various professional backgrounds, bureaucracy, financial institutions, think tanks, NGOs and academia (Anderson et al., 2020; Rozbicka & Spohr, 2016). The visible participants in agenda setting (such as politicians, elected officials, the media and decision makers) are often influenced by policy entrepreneurs who also raise public concern, come up with innovative solutions and ensure laws and policies are passed (Anderson et al., 2020). Policy windows opened by focussing events can be found on all jurisdictional levels. However, they differ in how each level conceptualises the issue onto the agenda and how long the policy window remains open (Michaels et al., 2006; Princen, 2007; Scholten, 2013). In the case of COVID-19, the policy window remains open although the urgency is starting to wane.

Policy entrepreneurs may not be the only ones to take advantage of policy windows, as there are questions around public influence during these opportunities. Barberá et al. (2019), for example, used Twitter data in their study to measure the amount of attention being paid to political issues and found that politicians rarely reflect the priorities of the general public. They argue that the general public, often the invisible participants, have a limited ability to influence the political agenda and that politicians are more likely to respond to their supporters.

While mainstream media tend to record and report, social media tend to critically examine announcements by decision makers, sometimes breaking the secrecy of political issues (Boynton & Richardson, 2016). Social media also have a role in revealing the use of placebo policies and non-action/non-decision making. Placebo policies are those that demonstrate government action over an issue, but whose true purpose is to distract from other agenda issues (McConnell, 2010, 2020). Placebo policies also mask potential interactions and interventions (see for example, Morrison et al., 2020). Policy windows can prompt non-action/non-decision making or, particularly during the COVID-19 Pandemic, the postponement of actioning an issue on the agenda. Non-action and postponement helps decision makers focus on the crisis at hand, which needs to take priority on the political agenda. Government non-decision making can also be the opportunity for industries and consumers to make their own decisions over an issue.

While social media may provide a powerful tool for individual voices, they do not necessarily mirror global opinion for individual voices. As social media have played an essential role in communicating health information (Tsao et al., 2021), it is not surprising to find that their use as a source of information is the top reason why 36% of consumers utilise them (Trifonova, 2020). The downside to this is that social media become fertile ground for misinformation. It is then essential for public health authorities to provide social media users with reliable scientific information to counter the spread of fake information (Hartley & Vu, 2020). Misinformation on social media is so common during health crises that the word infodemic has even been adopted (Zarocostas, 2020).

The relationship between information, social media and policy change has been marked by the Pandemic provoking increased time spent on social media. This context directly influenced the ‘plastic agenda’ which will be discussed in the following section.

### COVID-19 and the plastic agenda

The ‘plastic agenda’ refers to all issues relating to plastic creation, manufacturing, use, disposal, reuse and repurpose, and disposal that make it onto a political agenda. As of 2020, over 150 countries had enacted regulatory measures relating to single-use plastics and the majority of these related to the restriction and/or banning of plastic bags (da Costa et al., 2020). COVID-19 has severely slowed down the plastic agenda across the world. In some countries, this health crisis has led to the postponement or reversal of regulatory measures, laws and policies in response to managing the spread of the virus (da Costa, 2021; Silva et al., 2021).

Beyond the human and socio-economical cost, the COVID-19 Pandemic has marked a turning point for plastic pollution by inflating plastic waste quantities with the addition of COVID waste, consisting of single-use personal protective equipment (PPE), notably face masks and to a lesser extent rubber gloves (Ammendolia et al., 2021). In addition to this new waste, regional lockdowns and stricter hygiene practices entered daily life provoking an increase in the use of everyday single-use plastics such as shopping bags, coffee cups and take-away food containers (Parashar & Hait, 2021). As these changes occurred, the public reacted promptly on social media showing their comprehension, confusion and even frustration at the increase in plastic waste being generated and increasingly visible around the globe, on streets and sidewalks, in rivers and on beaches (e.g. Schofield et al., 2021).

Health and hygiene policies have resulted in the substantial increase of single-use PPE. While health and hygiene have been the main reasons governments have changed their plastic agendas, they are not the only reasons. Government decisions have also been driven by industry pressure, and the general politics that have arisen due to the window of opportunity provided by the Pandemic, all of which are faced with a diversity of consumer reactions especially when governments engage in non-decision making. Governments have also made decisions based on ‘crisis thinking’, where the focus is being prepared for and recovering from crises during regular, everyday policy making (Rhinard, 2019). Crisis thinking has resulted in the Pandemic being regarded by governments as an urgent crisis, while plastic pollution is seen as an on-going crisis (Vince, *in press*).

Industry pressure has heavily influenced agenda setting over the plastic pollution issue. The policy entrepreneurs who are driving corporate-friendly agendas and are engaged in ‘disaster lobbying’ use indicators such as COVID-19 statistics to show an increase of transmission and downplay the severity of pollution while stressing the hygiene/health benefits of single-use plastic (Johansson, 2021). Plastic industries saw this window of opportunity to ask for postponement of the ban on single-use plastics in Belgium (EUPC, 2020) and in the USA through the Plastic Industry Association (PIA). While the PIA argued that studies proved the risk that reusable bags may carry viruses and bacteria, health officials have stressed that there is a lack of scientific evidence to support this claim (da Costa, 2021).

Social media have contributed to raising awareness and making consumer demands heard during COVID-19. They also had a role in being a platform for information where new governmental decisions are shared. Consumer behaviour changed as a result of the health and hygiene concerns during the Pandemic with greater demand for banned single-use plastic products and for food packaging (Silva et al., 2020) that was driven by industry rather than government/regulation. The lack of government involvement and industry influence resulted in other changes in behaviour such as panic buying, stockpiling and online shopping at unprecedented rates (Parashar & Hait, 2021), all contributing to higher levels of plastic waste. Those shifts in consumer behaviour, as well as recommendations whether official or not, were recorded and commented upon on social media.

Within this policy context, little research has been done on how social media have impacted three key areas: industry pressure, consumer behaviour and politics. Social media are used here both as a tool to understand the relationship between the plastics agenda and these three key areas, and as a reflection of the facets of these relationships, offering insights into the application of these policies.

## Methodology

### Data gathering

As the use of social media has increased during the Pandemic (Sortlist, 2022), this provided a suitable archive from which to understand people’s perceptions of the ways in which COVID waste pollutes the environment and of policies influencing daily life. The three case studies included: the USA and the influence of industry pressure; Mexico and the politics behind the plastics agenda; and Australia and government non-decision making with regard to consumer behaviour. Amongst all social media, Twitter was selected as the ideal platform through which users discuss their feelings and reactions through tweets.^1^ Created in 2006, Twitter has over 353 million users per month (Dean, 2021) although its use varies greatly between countries. In the countries investigated, Twitter always ranks after YouTube as the favourite social media platform. For this investigation, data were retrieved through an Academic Twitter developer account and analysed in R.

Data analysis can be divided into three successive stages: (1) retrieving tweets, (2) cleaning the dataset and (3) analysing the dataset. Stage 1 involved investigating tweets using a Twitter developer account obtained for this academic project (https://developer.twitter.com/en/solutions/academic-research). This provided access to a full-archive search from the first tweet in 2006. To retrieve tweets, we used both the full-archive search (Australia and Mexico) and the user search (USA) available on the Academic TwitteR package on R (Barrie & Cho., 2021). The full-archive search looked for keywords specific to each policy while setting the time and space parameters to our case study (Table 1). We analysed results as aggregate data instead of direct quotes to ensure privacy of users. The user search compared the most commonly used words from two public and corporate accounts (Government of California and the American Chemistry Association) between 15 March and 31 July 2020.

**Table 1:** Summary of case studies and their associated methods for social media analysis

Case study: Policy responding to …	Policy	Country	Timeline	Words looked for
Industry pressure	Lift of plastic bag ban	USA California	Mid-March to end of July 2020	Not applicable as tweets were selected according to a user not a set of words
Politics	Ban on plastic bags	Mexico Mexico City	January to February 2020	ley de residuos sólidos OR bolsa de plástico Ciudad de México OR bolsa de plástico CDMX OR prohibición bolsa de plástico OR bolsa de plastico OR #bolsadeplastico OR #leyderesiduos
	Ban on single-use plastic		January to February 2021	ley de residuos sólidos OR plastico OR plasticos OR plástico OR plásticos OR plástico de un solo uso Ciudad de México OR plástico de un solo uso CDMX OR prohibición plástico de un solo uso
Responses to consumer behaviour	Ban of reusable cups	Australia	March 2020 to May 2021	Keep cup OR keep cups OR reusable coffee cup OR cafe reusable cup OR reusable coffee cups OR cafe reusable cups OR #keepcup OR #reusablecoffeecup

In Stage 2 all tweets were manually screened to ensure they were relevant to the topic discussed and to the policy investigated. Stage 3 involved Sentiment Analysis on R and Qualitative Analysis in NVivo. Tweets from the Australian example were analysed using sentiment analysis packages, such as Syuzhet (available on R), and following the code written by Yanqing Shen (2020). This enabled a comparison through time of the sentiments associated with reusable cups and to understand whether this has shifted as a result of the Pandemic, and the non-action of the Australian Government. The qualitative analysis in NVivo 20 involved analysing tweets from the US and Mexican case studies, respectively, providing an understanding of the occurrence of terms and general themes emerging from the tweets. Search of specific terms (e.g. looking for mentions of plastics) and frequencies of most common words used (in the form of word clouds) were undertaken to analyse resemblance of discourses in the USA, while coding was undertaken for the Mexican case study. Thematic analysis of the tweets’ content allowed the identification of emerging themes (Fereday & Muir-Cochrane, 2006: 82) through familiarity with the data (see Savin-Baden & Howell Major 2013, Chapter 28). Results are presented as a discussion of vocabulary, themes and feelings emerging from the tweets in the form of aggregate data. Ethical clearance was obtained for this research through the University of York’s Department of Archaeology.

## Results

### Industry pressure in the USA

In the case of industry pressure, we specifically looked at the state of California where we focussed on the type of communication offered by the government and the American Chemistry Association (AmChem). In the USA, in 2020, there was a postponement of plastic bag bans in several states. California suspended the ban in April 2020 but reinstated it after 2 months, offering a small time frame for the window of opportunity to be analysed. The goal of this case study was to understand the type of communication surrounding the rise of the Pandemic and the suspension of bans along with industry pressure. To do so, official Twitter communications by the Office of the Governor of California were analysed around changes due to the Pandemic, such as the lift of the plastic ban by Gavin Newsom in California (Paragraph 12 of Executive Order N-54-20) and its reintroduction 60 days later. The hypothesis was that governments were likely to use the same hygienist arguments used by plastic industries, perhaps ceding to industry pressures.

**Fig. 1:**
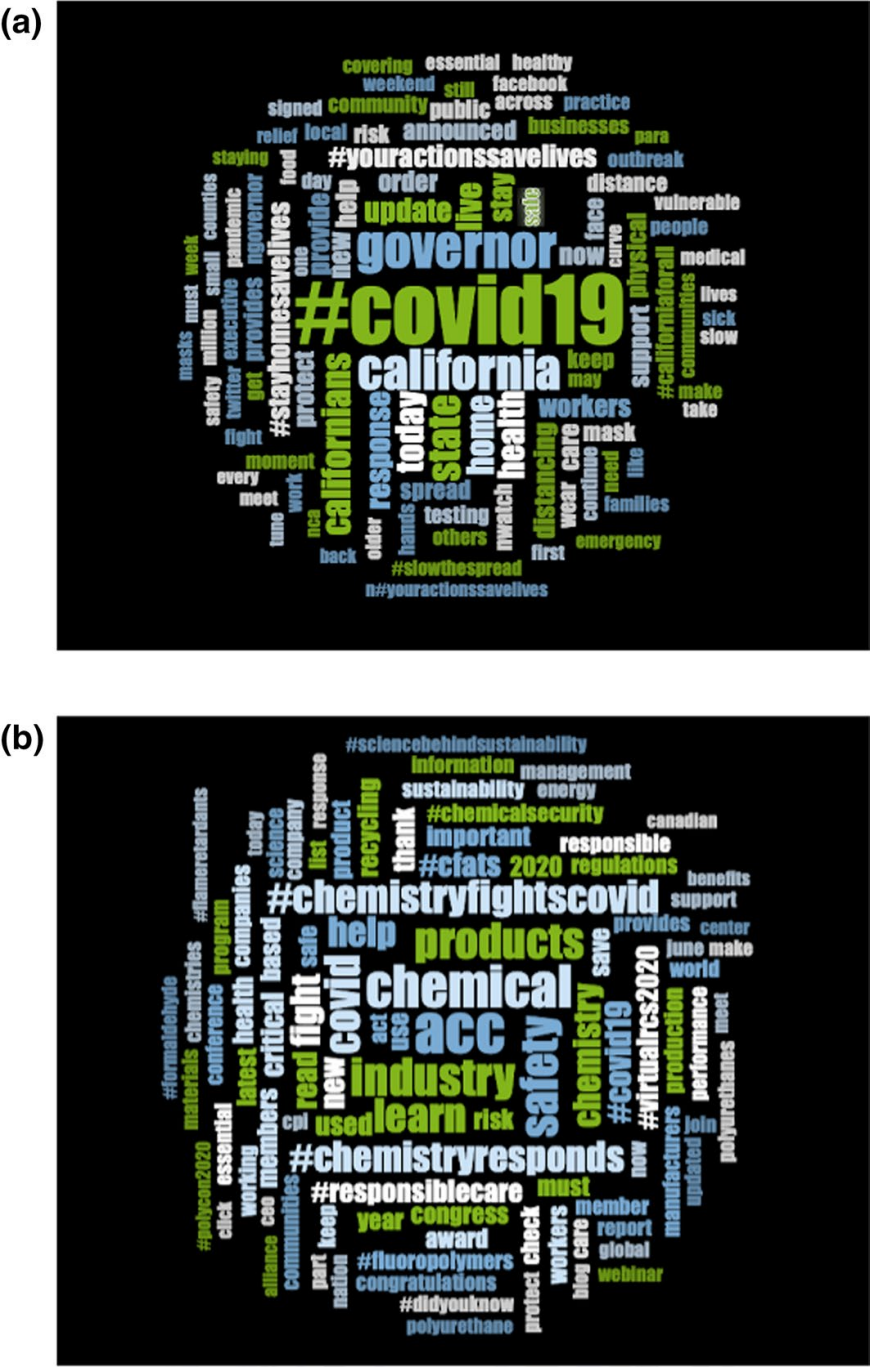
**a** Wordcloud of most common terms used in the tweets published by the Government of California between mid-March to end of July 2020. **b** Wordcloud of most common terms used in the tweets published by the American Chemistry Association between mid-March to end of July 2020

We undertook a user search approach, which only considered original tweets, discounting manually the retweets^2^ as no functionality exists for that in the Academic TwitteR package. This left 1026 tweets and 253 tweets for the Government of California and AmChem, respectively. The vocabulary used was analysed for both accounts through NVivo functionalities of word clouds and word search (Fig. 1). The aim was to see whether and how policies regarding a lifting of plastic bag bans were discussed.

Surprisingly, the word ‘plastic’ was not even used in government tweets and the communication focuses more on sharing feelings such as safety, community and a sense of response (Fig. 1a). No communication evidenced the suspension of the plastic ban as the focus was shifted towards a semantic field reinforcing the emergency of the COVID-19 situation and the sense of community and responsibility needed to face it. This is quite similar to the type of language used at the outbreak of the Pandemic by plastic industries: ‘Safety’, ‘Help’, ‘Fight’, 'Risk’ and ‘Responsibility’ are amongst the recurrent terms used by AmChem (Fig. 1b).

Those terms provide a sense of safety to consumers and appear to draw attention away from the environmental impact of plastic bans. Here, the window of opportunity is associated with communication emphasising the sense of crisis. In that way, the classification of the discourse puts forward priority of the circumstances while completely ignoring a ban that impacted producers, consumers and the environment. Rather, AmChem is presented as an ally during the fight against the virus, contributing to this semantic association between plastic and safety.

### Politics in Mexico

In the Mexico City case study, we sought to understand how people perceived, over 2 months after their introduction, the two policies regarding plastic use that were implemented during the Pandemic: the plastic bag ban in Mexico City in January 2020 and the single-use plastic ban in January 2021. In total, 93 tweets were examined related to the ban on plastic bags in 2020 and 43 tweets related to the single-use plastic ban in 2021. The plastic item bans were a contrast to other government policies of President Andrés Manuel Lopez Obrador (AMLO) where the environment was not a priority (e.g. favouring the construction of oil refineries, a mega railway project in the Maya region threatening the biodiversity and heritage of indigenous communities, and a new airport in Mexico City). With this example, our hypothesis was that social media would reflect the level (or lack) of trust in the measures taken. In Mexico City, plastic bags were banned from supermarkets in January 2020 (Secretaria del Medio Ambiente, 2021). As of 1 January 2021, Mexico City reaffirmed its position by ensuring the ban on selling, commercialising and distributing single-use plastic items, such as straws, plastic cups, tampon applicators and plastic cutlery (Secretaria del Medio Ambiente, 2021). Those bans were, respectively, followed by queries from the Asociación Nacional de la Industria de Plásticos (ANIPAC) to postpone those laws due to the Pandemic invoking similar actions in the USA and in European countries (ANIPAC, 2020) and by severe critiques due to the impact it would have on employment, with a loss of approximately 50,000 jobs (Stettin & Ordaz, 2021). While the bans on plastic bags and single-use plastics were implemented in Mexico City, they were postponed in several states of the country, such as Oaxaca, Nayarit and Acapulco, following recommendations from plastic industries (Olivera, 2020).

Thematic coding revealed two recurring themes in tweets reacting to the ban of plastic bags in Mexico City in 2020. First, a third of the tweets referred to the application of the law. Twenty tweets reveal information regarding the compliance (*n* = 12) or the disobedience (*n* = 8) to the measure by establishments. Concerns regarding the application of the measure also appear in the tweets, questioning the financial benefits for the companies and asking about fines. Tweets also question the policy application without alternatives being given to the consumers. Second, 60% of tweets focus on the emotional response that can be categorised into three types: (1) People can have a positive perception of the law: they consider it as a great step for the environment. (2) By contrast, some tweets are negative, claiming that the measure is a mistake for a variety of reasons: the greater energetic investment required for the production of paper bags; the consequential loss of jobs in plastic industries; and the hidden financial benefits of supermarkets being able to charge for paper bags. (3) Several tweets are written in an ironic tone using references to the paradox of the law with the real problem lying in recycling and packaging. Several tweets actually stand against the measure (*n* = 15), but positive reactions were more common (*n* = 23) along with the ironic statements (*n* = 18). The global environmental impact of plastic bags is also discussed by several users and contrasts with the environmental cost of paper bags.

**Fig. 2:**
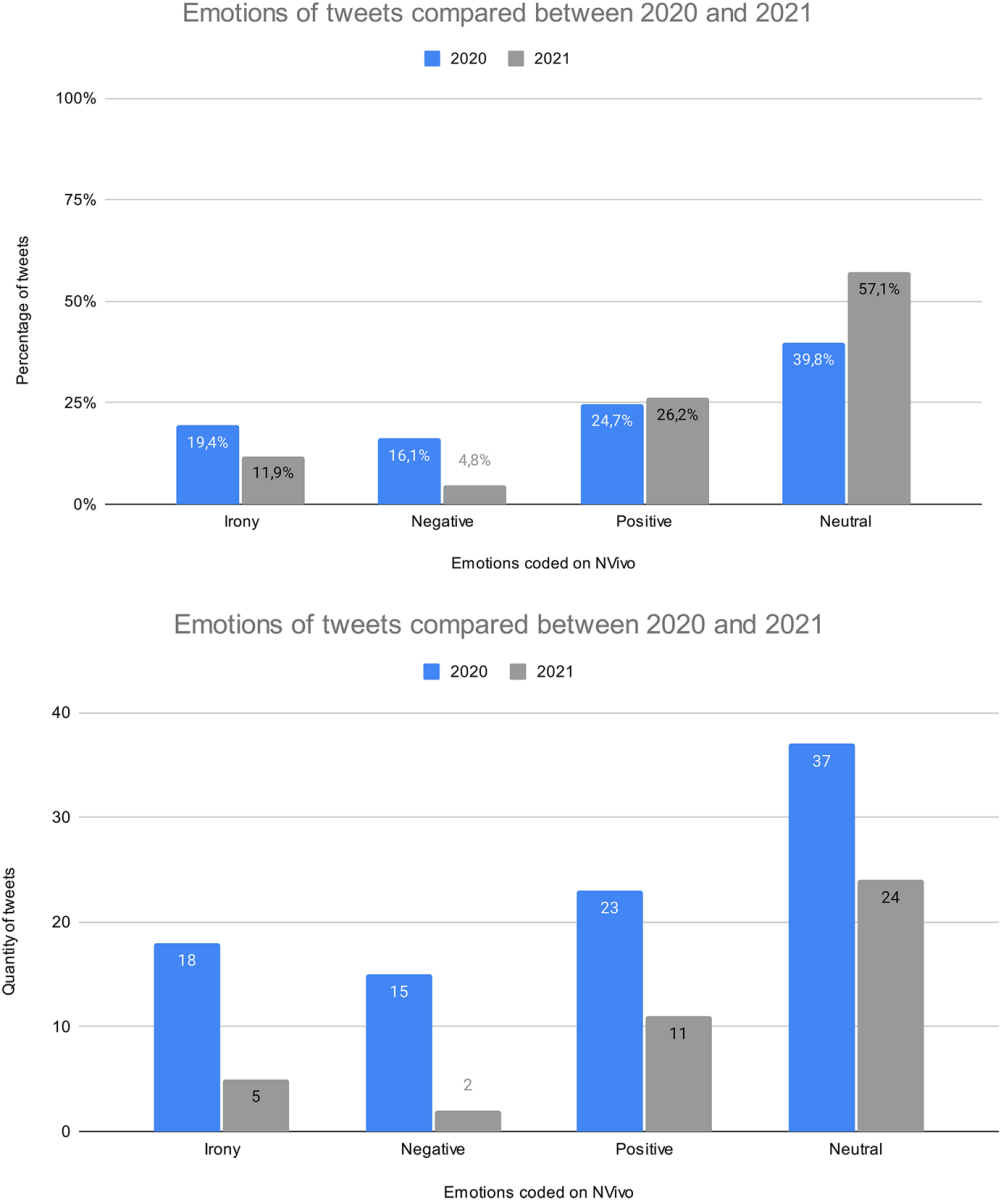
Comparison of tweets related to the plastic bag and single-use plastic ban in Mexico in 2020 and 2021, respectively

Tweets in 2021 targeted several topics, notably the lack of decision making based on scientific data, the questionable measurements used and the lack of proposed alternatives. For instance, users questioned the next steps as styrofoam was not banned by the plastics policies. Reactions reflected a specific concern with take-away packaging, and menstrual sanitary products (since tampons with plastic applicators were banned), emphasising the lack of alternatives. Several users also condemned the policy as a decision that was not thought through and that took advantage of a trending issue. Overall, this policy on plastic appeared to have provoked less comments in 2021 compared to 2020 (Fig. 2) and less emotional reaction with only 43% of tweets expressing either negative, positive or ironic responses, the rest comprising tweets that share the news in a neutral way, either asking for people’s opinion or sharing resources.

### Consumer behaviour in Australia

Finally, we wanted to also investigate the reaction of Twitter users to the ban on reusable plastic cups in coffee shops in Australia and how this evolved through the Pandemic. Prior to the Pandemic and on the back of the ABC documentary “War on Waste”, the uptake of reusable coffee cups had reached over 40% (Barnfield & Marks, 2017). However, during the Pandemic, reusable coffee cups and other containers were no longer being accepted by providers due to hygiene concerns, causing a shift back towards single-use plastic items (Sandhu et al., 2021). We examined this situation by dividing the Pandemic in Australia into three time periods:

- 1 March 2020–31 July 2020—Start of the Pandemic and nationwide lockdown, peak of the second wave
- 1 August 2020–31 December 2020—Recovery from the second wave and opening up of many states and the sense of normality returning
- 1 January 2021–31 May 2021—The start to a relatively COVID-19-free year, prior to the return of lockdowns caused by the Delta strain in July 2021 within the two most populous states of New South Wales (NSW) and Victoria.

KeepCup is an Australian brand that paved the way for using reusable cups in coffee shops. Its popularity turned it into a proprietary eponym in Australia when referring to this type of product. During the Pandemic, this tendency was abruptly stopped by bans of reusable cups in cafes and takeaway shops (Smith, 2020). The aim here was to understand whether the health/hygiene argument emerged from changing psychology in consumer behaviour or whether it was used as an excuse to re-introduce plastics following the pressure of industries. The hypothesis was that consumer behaviours changed to include more single-use plastic as coffee shops were reluctant to accept reusable cups, and that these behaviour changes were due to perceived health and safety risks, rather than a demand from consumers. Interestingly, there was no mandatory policy obliging coffee-shop owners to serve drinks in disposable cups, yet it became widespread practice during the Pandemic (The State of Victoria Department of Environment Land Water & Planning, 2020).

**Fig. 3:**
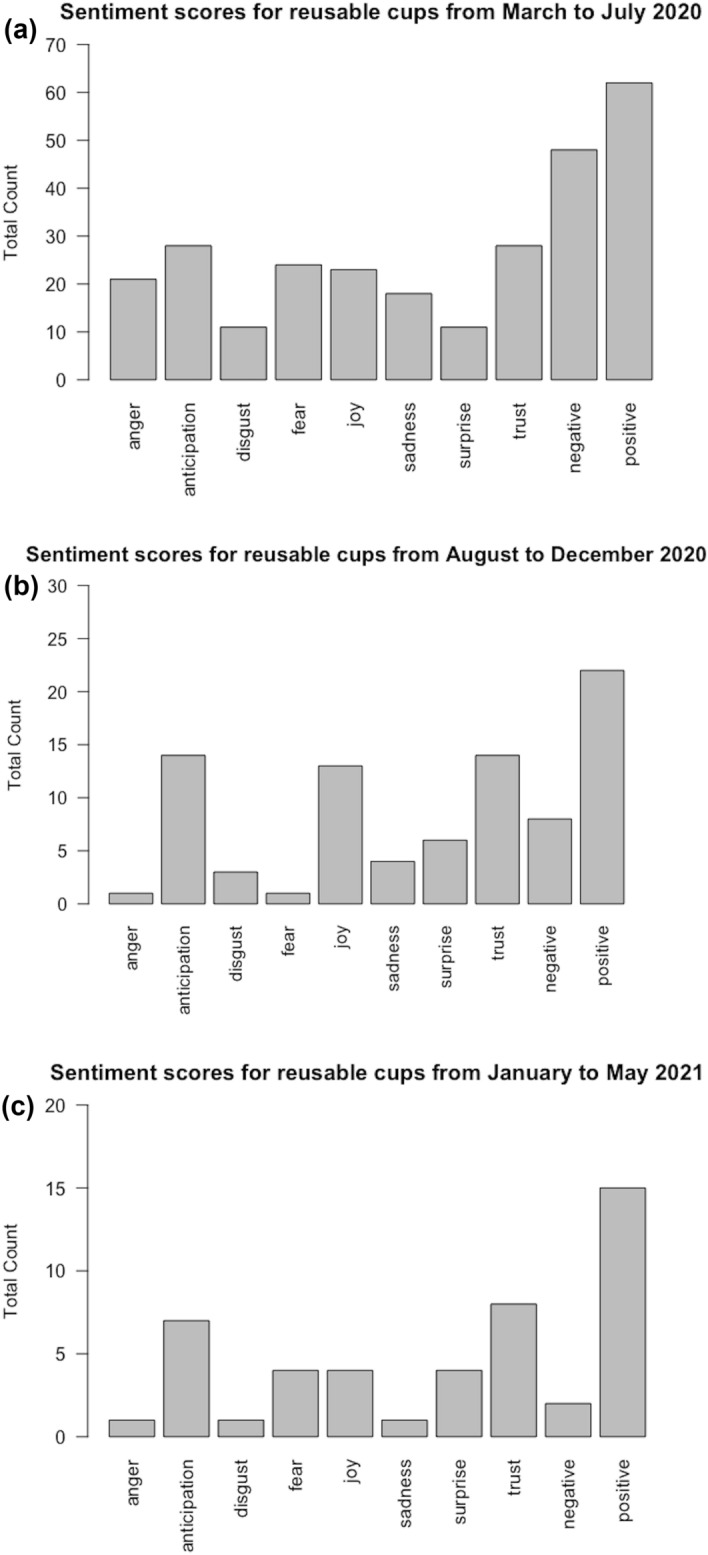
Sentiment score analysis for reusable cups between **a** March and July 2020, **b** August and December 2020 and **c** January to May 2021

Tweets were examined relating to the use of reusable cups in cafes throughout this period. Sentiment analysis undertaken in R (Fig. 3 before manual data cleaning), placed emphasis on the prevalence of positive sentiments associated with the words “keep cup” and “reusables”. Keep cups were still perceived positively even during the Pandemic. To gain a better idea of content, manual data cleaning identified tweets directly related to the use of keep cups in cafés. With a sample of 55, 23 and 9 tweets for each period, respectively, analysis of content was undertaken. Although the number of tweets is small, the impression, on closer inspection, is that there was disagreement amongst customers with the temporary ban of reusable cups in cafes in the first period. Some users were more vehement than others and most seemed to accept the situation with disappointment. Only a few Twitter users seemed to value this decision (*n* = 6). During the following periods, there was a decrease of interest for the topic marked by the reduced number of relevant tweets (as stated above: 59 tweets in Period 1, 23 in Period 2 and 9 in Period 3). This is also visible in the sentiment analysis before selection of directly relevant tweets (Fig. 3). Period 2 is associated more with questions on whether keep cups were going to be allowed. It also appeared that some users were finally able to go back to their old sustainable habits. Period 3 saw a drastic fall in tweets related to keep cups. One user noted that people reverted to the use of disposable cups, whereas other users commented on their use of reusable cups in their local cafes.

Although the numbers are small, these results appear to suggest that throughout the entire time span people continued to perceive reusable cups in a positive manner. This is also suggested by the general positive feeling evident in the sentiment analysis of tweets including the word reusable or keep cups.

## Discussion

The three case studies demonstrate the diversity of approaches for social media analysis to consider the relationships between people and agenda setting. Social media can serve as a platform to send reassuring messages in times of crisis while aligning with industry discourse on health and hygiene questions (US case study). But social media can also be used by people to show their (dis)agreement with new policies and to question their design (Mexican case study). Consumers can also indicate a relatively stable positive attitude towards their eco-friendly behaviours despite the lack of government action (Australian case study).

In the US case study our hypothesis was that governments would use the same hygienist arguments used by plastic industries, perhaps ceding to industry pressures. The plastic industry and pro-plastic entrepreneurs engaged in disaster lobbying to change political agendas. The plastic industry entrepreneurs in the US case study who were once invisible during peak plastic usage, had become visible during the Pandemic. They utilised all agenda-setting opportunities to leverage their case—through the use of COVID-19 as a focussing event to increase the size of the policy window to postpone the implementation of plastic policies.

In the Mexico City case study, we hypothesised that the plastic ban policies did not reflect the greater political agenda. This lack of consistency contributed to mistrust of the government, which was reflected in the social media analysis. In 2020, 44% of tweets about plastic were negative. Those tweeting, often invisible participants in the policy process, also commented on the lack of plastic alternatives and the government’s poor environmental policy track record. In this case, numerous tweets reflect that the plastic bans were considered ineffective to tackle plastic pollution and were therefore only placebo policies that appear to solve an issue but have minimal impact. Decision makers needed to acknowledge the political context where the dominance of legislatures, political parties, interest groups/entrepreneurs and public opinion differ (Sanjurjo, 2020). The case study therefore demonstrates the importance of putting into context the plastics policies and decisions within the broader political landscape and agenda that they belong to, not least because policies can be presented as placebo within a broader political agenda.

In the Australian example, we hypothesised that despite stable consumer behaviour perceiving the reusable cups positively throughout the Pandemic, the usage of single-use plastics increased as coffee shops were reluctant to accept reusable cups due to perceived health and safety risks. The crisis thinking characterising the first months of the Pandemic may have led to decisions that were protecting business interests and not necessarily reflecting people’s perception of keeping cups and reusables during this time. This may have led to the decision to ban reusable cups in a moment where concerns for hygiene and limitation of contact were essential strategies by industry. Although our conclusions cannot be generalised due to the small sample size, the results might suggest that the Australian federal and state governments engaged in non-decision making, allowing industry to self-regulate. Notably, political agendas were dominated by mandates on PPEs and some other forms of health-related COVID plastics, but not single-use items such as coffee cups.

Across the three case studies, decision makers needed to drive the plastic problem as an urgent, rather than a creeping crisis, whether alongside COVID-19 or as a separate crisis exacerbated by it. If done strategically and cautiously, it could have resulted in positive policy change that could have reduced, rather than increased, plastic pollution.

## Conclusion

In this paper we describe and test an innovative approach to exploring the potential of social media analysis to investigate ways that environmental crises such as plastic pollution can be manipulated by governments and industry, but which can also be used by entrepreneurs and invisible participants to react and (dis)approve new decisions in plastics policy making. Policy windows and focussing events such as the COVID-19 Pandemic often bring to the surface opportunities for crisis lobbying and to shift issues on or off the political agenda. However, as plastics policies return to political agendas due to the mounting of COVID and other plastic waste across the world and policy reversals are reinstated, entrepreneurs interested in reducing plastic pollution are becoming more visible once again. The political motivation needed to activate crisis thinking over plastics policies could be beneficial in resetting the single-use plastics agenda.

The proof of concept demonstrated here suggests that systematic social media analysis could have a wider application in understanding decisions and reactions to other political issues dominating agendas.

## Footnotes

1 We here understand tweets as each comprising a “short message also known as a post, status or microblog from a user on Twitter and which consists of a < 140 characters” (Ahmed et al., 2017: 4).

2 “The retweet function forwards a tweet from a user to their followers” (Ahmed et al., 2014: 4). We here decided not to consider retweets as they create doublons.
